# Supplementary material for: Biodiversity of ecosystems in an arid setting: The late Albian plant communities and associated biota from eastern Iberia
Source: PLoS One. 2023 Mar 2;18(3):e0282178. doi: 10.1371/journal.pone.0282178 (PMC9980801; doi:10.1371/journal.pone.0282178)
Supplement: S2 Appendix — (DOC) [file pone.0282178.s002.doc]

**Appendix 2_** List of the identified taxa ordered according their botanical affinity and zoological identification and references of the taxonomic authorities

**Aquatic palynomorphs**

Freshwater algae

*Botryococcus braunii* Kutzing, 1849 (AP, CA and SJ sections)

*Chomotriletes minor* (Kedves, 1961) Pocock, 1970 (AP, CA and SJ sections)

*Ovoidites* sp. (CA section)

*Tetraporina* sp. (SJ section)

Prasinophytes

*Cymathiosphaera* spp. (AP and CA sections)

*Schizosporis reticulatus* Cookson and Dettman, 1959 (AP and CA sections)

Tasmanaceae? (AP section)

*Tasmanites* sp. (CA section)

Dinoflagellate cysts

*?Apteodinium* sp. (CA section)

*?Callaiosphaeridium assymetricum* (Deflandre and Courteville, 1939) Davey and Williams, 1966 (AP section)

*?Chichaouadinium arabicum* Below, 1981 (SJ section)

*Chlamydophorella* sp. (CA section)

*Circulodinium brevispinosum* (Millioud, 1969) Fauconnier in Fauconnienr and Masure, 2004 (CA section)

*Cometodinium* sp. (CA section)

*Cribroperidinium* spp. (AP section)

*Cyclonephelium chabacca* Below, 1981 (AP and CA sections)

*Cyclonephelium vannophorum* Davey, 1969 (CA section)

*Cyclonephelium* spp. (AP, CA and SJ sections)

*Dapsilidinium* sp. (SJ section)

*Exochosphaeridium* sp. (AP section)

*Florentinia mantellii* (Davey and Williams, 1966) Davey and Verdier, 1973 (AP and SJ sections)

*Florentinia* sp. (CA section)

*Ginginodinium* cf. *evittii* Singh, 1983 (SJ section)

*Impletosphaeridium* spp. (AP, CA and SJ sections)

?*Kallosphaeridium* spp. (SJ section)

*Kiokansium unituberculatum* (Tasch, 1964) Stover and Evitt, 1978 (AP and CA sections)

*Odontochitina rakodes* Bint, 1986 (SJ section)

*Oligosphaeridium complex* (White, 1842) Davey and Williams, 1966 (AP, CA and SJ sections)

*Oligosphaeridium* spp. (CA and SJ sections)

*Ovoidinium* sp. (CA section)

*Palaeohystrichophora* sp. (SJ section)

*Palaeoperidinium cretaceum* (Pocock, 1962) Lentin and Williams, 1976 (SJ section)

*Spiniferites twistring*iensis (Maier, 1959) Fensome et al., 1990 (CA section)

*Spiniferites* spp. (CA and SJ sections)

*Subtilisphaera* sp. (CA section)

*Tehamadinium* sp. (CA section)

*Tenua hystrix* Eisenack, 1958 (SJ section)

*Trichodinium castanea* Deflandre, 1935 ex Clarke and Verdier, 1967 (AP and CA sections)

Acritarchs

*Micrhystridium* sp. (AP, CA and SJ sections)

Other aquatic palynomorphs

Lining of Foraminifera (SJ section)

**Spores**

Hepaticophyta

*Aequitriradites spinulosus* (Cookson and Dettmann, 1958) Cookson and Dettmann,1961 (CA section)

*Aequitriradites* sp. (AP section)

*Couperisporites* sp. (AP section)

*Foraminisporis asymmetricus* (Cookson and Dettman, 1958) Dettman, 1963 (AP, CA and SJ sections)

*Foraminisporis* cf. *undulatus* Leffingwell, 1971 (CA section)

*Foraminisporis* sp. (CA section)

*Triporoletes cenomanianus* (Agasie, 1969) Srivastava, 1975 (CA section)

*Triporoletes radiatus* (Dettmann, 1963) Playford, 1971 (CA section)

*Triporoletes reticulatus* (Pocock,1962) Playford, 1971 (CA section)

*Triporoletes simplex* (Cookson and Dettmann, 1958) Playford, 1971 (AP section)

*Triporoletes* sp. (CA and SJ sections)

Bryophyta

*Antulsporites clavus* (Balme, 1953) Filatoff, 1975 (SJ section)

cf. *Antulsporites* sp. (AP section)

*Cingutriletes clavus* (Balme, 1953) Dettmann, 1963 (CA section)

*Cingutriletes congruens* Pierce, 1961 (CA and SJ sections)

*Cingutriletes* spp. (AP and CA sections)

*Nevesisporites dailyi* (Cookson and Dettmann, 1958) Backhouse, 1988 (AP, CA and SJ sections)

*Polycingulatisporites reduncus* (Bolkhovitina, 1953) Playford and Dettmann, 1965 (AP and CA sections)

*Stereisporites psilatus* (Ross, 1949) Thomson and Pflug, 1953 (SJ and CA sections)

*Stereisporites* spp. (AP, CA and SJ sections)

*Taurocusporites segmentatus* Stover, 1962 (AP, CA and SJ sections)

*Taurocusporites* sp. (AP and CA sections)

Lycophyta

*Camarozonosporites ambigens* (Fradkina, 1964) Playford, 1971 (CA section)

*Camarozonosporites* cf. *ambigens* (Fradkina, 1964) Playford, 1971 (CA section)

*Camarozonosporites* cf. *hammenii* Van Ameron, 1965 (CA section)

*Camarozonosporites insignis* Norris, 1967 (AP section)

*Camarozonosporites* spp. (AP and CA sections)

*Ceratosporites* sp. (CA and SJ sections)

*Densoisporites velatus* Weyland and Krieger, 1953 (CA section)

*Densoisporites* sp. (AP and SJ sections)

*Echinatisporis varispinosus* (Pocock, 1962) Srivastava, 1975 (SJ section)

*Echinatisporis* sp. (AP and CA sections)

*Foveosporites* cf. *subtriangularis* (Brenner, 1963) Döring, 1965 (AP and CA sections)

*Kraeuselisporites* sp. (= *Patellasporites? echinatus* Groot and Groot, 1962) (SJ section)

*Kraeuselisporites* spp. (AP and CA sections)

*Lycopodiumsporites* sp. (AP section)

*Neoraistrickia* cf. *densata* Filatoff, 1975 (CA section)

*Neoraistrickia equalis* (Cookson and Dettmann, 1958) Backhouse, 1988 (CA section)

*Neoraistrickia robusta* Brenner, 1963 (AP, CA and SJ sections)

*Neoraistrickia truncata* (Cookson, 1953) Potonié, 1956 (AP and CA sections)

*Neoraistrickia* spp. (AP, CA and SJ sections)

*Retitriletes austroclavatidites* (Cookson, 1953) Döring, Mai, Krutzsch and Schulz in Krutzsch, 1963 (AP and CA sections)

*Retitriletes* *clavatoides* (Couper, 1958) Döring, Mai, Krutzsch and Schulz in Krutzsch, 1963 (AP section)

*Retitriletes* sp. (AP, CA and SJ sections)

*Sestrosporites pseudoalveolatus* (Couper, 1958) Dettmann, 1963 (CA section)

*Staplinisporites caminus* (Balme, 1957) Pocock, 1962 (AP and SJ sections)

*Staplinisporites* cf. *telatus* (Balme, 1957) Döring, 1965 (AP section)

*Uvaesporites* sp. (CA section)

Monilophyta Anemiaceae

*Appendicisporites bifurcatus* Singh, 1964 (AP section)

*Appendicisporites bilateralis* Singh, 1971 (AP, CA and SJ sections)

*Appendicisporites* cf. *concentricus* Kemp, 1970 (AP and CA sections)

*Appendicisporites* cf. *crenimurus* Srivastava, 1972 (CA section)

*Appendicisporites cristatus* (Markova, 1961) Pocock, 1964 (CA section)

*Appendicisporites* *erdtmanii* Pocock, 1964 (AP and CA sections)

*Appendicisporites* *fucosus* Vavrdov, 1964 (AP and CA sections)

*Appendicisporites* cf. *fucosus* Vavrdov, 1964 (SJ section)

*Appendicisporites* cf. *jansonii* Pocock, 1962 (AP and CA sections)

*Appendicisporites potomacensis* Brenner, 1963 (CA section)

*Appendicisporites* cf. *potomacensis* Brenner, 1963 (AP section)

*Appendicisporites problematicus* (Burger, 1966) Singh, 1971 (AP section)

*Appendicisporites* cf. *problematicus* (Burger, 1966) Singh, 1971 (AP and CA sections)

*Appendicisporites robustus* Kemp, 1970 (CA section)

*Appendicisporites tricornitatus* Weyland and Greifeld, 1953 (AP and CA sections)

*Appendicisporites* cf. *tricornitatus* Weyland and Greifeld, 1953 (CA section)

*Appendicisporites* spp. (AP, CA and SJ sections)

*Cicatricosisporites angicanalis* Döring, 1965 (CA section)

*Cicatricosisporites apicanalis* Paden Phillips and Felix, 1972 (AP, CA and SJ sections)

*Cicatricosisporites aralicus* (Bolkhovitina, 1961) Brenner, 1963 (AP and SJ sections)

*Cicatricosisporites crassiterminatus* Hedlund, 1966 (CA section)

*Cicatricosisporites dorogensis* Potonié and Gelletich, 1933 (CA section)

*Cicatricosisporites* cf. *grabowensis* Döring, 1965 (SJ section)

*Cicatricosisporites hughesii* Dettmann, 1963 (AP, CA and SJ sections)

*Cicatricosisporites* cf. *ludbrookiae* Dettmann, 1963 (AP section)

*Cicatricosisporites* cf. *minutaestriatus* (Bolkhovitina, 1961) Pocock, 1964 (AP section)

*Cicatricosisporites patapscoensis* Brenner, 1963 (AP, CA and SJ sections)

*Cicatricosisporites potomacensis* Brenner, 1963 (AP, CA and SJ sections)

*Cicatricosisporites proxiradiatus* Kemp, 1970 (CA section)

*Cicatricosisporites* *pseudotripartitus* (Bolkhovitina, 1961) Dettmann, 1963 (AP section)

*Cicatricosisporites* cf. *pseudotripartitus* (Bolkhovitina, 1961) Dettmann, 1963 (AP, CA and SJ sections)

*Cicatricosisporites* cf. *recticicatricosus* Döring, 1965 (CA section)

*Cicatricosisporites sinuosus* Hunt, 1985 (AP and CA sections)

*Cicatricosisporites sprumontii* Döring, 1965 (CA section)

*Cicatricosisporites venustus* Deák, 1963 (B, CA and SJ sections)

*Cicatricosisporites* spp. (AP, CA and SJ sections)

*Cicatricososporites auritus* Singh, 1971 (CA section)

*Cicatricososporites phaseolus* (Delcourt and Sprumont, 1955) Krutzsch, 1959 (CA section)

*Costatoperforosporites fistulosus* Deák, 1962 (AP and CA sections)

*Costatoperforosporites foveolatus* Deák, 1962 (AP, CA and SJ sections)

*Costatoperforosporites triangulatus* Deák, 1962 (AP and CA sections)

*Costatoperforosporites* cf.  *triangulatus* Deák, 1962 (SJ section)

*Costatoperforosporites* spp. (AP, CA and SJ sections)

*Nodosisporites segmentus* (Brenner, 1963) Davies, 1985 (AP section)

*Nodosisporites* sp. (CA section)

*Ruffordiaspora* sp. (AP and CA sections)

Monilophyta Botryopteridales

*Granulatisporites infirmus* (Balme, 1957) Cornet and Traverse, 1975 (CA section)

*Granulatisporites michinus* (Srivastava, 1975) Ravn and Witzke, 1995 (CA section)

*Granulatisporites* sp. (AP and CA sections)

*Lophotriletes* sp. (AP section)

Monilophyta Dipteridaceae/Matoniaceae

*Dictyophyllidites equiexinus* (Couper, 1958) Dettmann, 1963 (AP and CA sections)

*Dictyophyllidites harrisii* Couper, 1958 (AP, CA and SJ sections)

*Dictyophyllidites* sp. (CA section)

*Matonisporites crassiangulatus* (Balme, 1957) Dettmann, 1963 (CA section)

*Matonisporites* sp. (AP section)

*Phanerosorisporites surensis* Juhász, 1979a (CA section)

*Phanerosorisporites*? sp. (AP section)

Monilophyta Gleicheniaceae

*Clavifera triplex* (Bolkhovitina, 1953) Bolkhovitina, 1966 (CA section)

*Clavifera* sp. (CA and SJ sections)

*Gleicheniidites* cf. *bolchovitinae* Döring, 1965 (SJ section)

*Gleicheniidites carinatus* (Bolchivitina, 1953) Bolchovitina, 1968 (CA section)

*Gleicheniidites laetus* (Bolchovitina, 1953) Bolchovitina, 1968 (SJ section)

*Gleicheniidites senonicus* Ross, 1949 emend. Skarby, 1964 (AP, CA and SJ sections)

*Gleicheniidites umbonatus* (Bolchovitina, 1953) Bolchovitina, 1968 (CA section)

*Ornamentifera peregrina* (Bolchovitina, 1953) Bolchovitina, 1968 (CA and SJ sections)

*Ornamentifera* cf. *tuberculata* (Grigorjeva, 1961) Bolchovitina, 1968 (CA section)

*Ornamentifera* sp. (AP and CA sections)

*Plicifera* sp. (AP and CA sections)

Monilophyta Lygodiaceae/?Dicksoniaceae

*Concavissimisporites* cf. *crassatus* (Delcourt and Sprumont, 1955) Delcourt, Dettmann and Hughes, 1963 (AP, CA and SJ sections)

*Concavissimisporites* *granulatus* Pocock, 1964 (AP and CA sections)

*Concavissimisporites* cf. *irregularis* (Pocock, 1970) Backhouse, 1988 (AP and SJ sections)

*Concavissimisporites punctatus* (Delcourt and Sprumont, 1955) Brenner, 1963 (CA section)

*Concavissimisporites verrucosus* Delcourt & Sprumont, 1955 emend. Delcourt, Dettmann and Hughes, 1963 (CA section)

*Concavissimisporites* spp. (AP, CA and SJ sections)

*Impardecispora* cf. *apiverrucata* (Couper, 1958) Venkatachala, Kar and Raza, 1969 (CA section)

*Trilobosporites* spp. (AP, CA and SJ sections)

Monilophyta Osmundaceae

*Baculatisporites comaumensis* (Cookson, 1953) Potonié, 1956 (AP, CA and SJ sections)

*Baculatisporites* sp. (AP and CA sections)

*Osmundacidites wellmanii* Couper, 1953 (AP, CA and SJ sections)

*Punctatisporites couperi* Ravn, 1995 (CA section)

*Rotverrusporites* sp. (CA section)

Monilophyta ?Polypodiaceae

*Laevigatosporites ovatus* Wilson and Webster, 1946 (AP and CA sections)

*Laevigatosporites* spp. (CA and SJ sections)

Monilophyta Pteridaceae

*Contignisporites* *cooksoniae* (Balme, 1957) Dettmann, 1963 (AP and SJ sections)

*Contignisporites* cf. *cooksoniae* (Balme, 1957) Dettmann, 1963 (CA section)

*Contignisporites* sp. (AP and CA sections)

Monilophyta Salviniales/?Marsileaceae

*Crybelosporites pannuceus* (Brenner, 1963) Srivastava, 1975 (AP, CA and SJ sections)

Monilophyta Schizaeaceae

*Distaltriangulisporites irregularis* Singh, 1971 (CA section)

*Distaltriangulisporites* *maximus* Singh, 1971 (CA section)

*Distaltriangulisporites* cf. *maximus* Singh, 1971 (CA section)

*Distaltriangulisporites perplexus* (Singh, 1964) Singh, 1971 (SJ section)

*Distaltriangulisporites* cf. *perplexus* (Singh, 1964) Singh, 1971 (CA section)

*Distaltriangulisporites* sp. (CA section)

*Ischyosporites* *crateris* Balme, 1957 (CA section)

*Ischyosporites* cf. *pseudoreticulatus* (Couper, 1958) Döring, 1965 (AP section)

*Ischyosporites* cf. *punctatus* Cookson and Dettmann, 1958 (CA section)

*Ischyosporites variegatus* (Couper, 1958) Schulz, 1967 (CA section)

*Ischyosporites* sp. (CA and SJ sections)

Monilophyta unknow affinities

*Acritosporites* cf. *kyrtomus* Juhász, 1979b (CA section)

*Acritosporites* sp. (SJ section)

*Biretisporites potoniaei* Delcourt and Sprumont, 1955 (AP and CA sections)

*Biretisporites* spp. (CA and SJ sections)

*Cardioangulina* sp. (CA section)

*Cibotiumspora jurienensis* (Balme, 1957) Filatoff, 1975 (AP and CA sections)

*Cibotiumspora* sp. (SJ section)

*Converrucosisporites geniculatus* Deák and Combaz, 1967 (AP and SJ sections)

aff. *Converrucosisporites kopukuensis* (Couper, 1953) Stover and Partridge, 1973 (AP and CA sections)

*Converrucosisporites* spp. (AP and CA sections)

*Cyathidites australis* Couper, 1953 (AP, CA and SJ sections)

*Cyathidites minor* Couper, 1953 (AP, CA and SJ sections)

*Deltoidospora* spp. (AP, CA and SJ sections)

*Distaverrusporites* sp. (AP section)

*Foveotriletes* cf. *parviretus* (Balme, 1957) Dettmann, 1963 (AP and CA sections)

*Gregussisporites orientalis* Juhász and Smirnova, 1985 (AP, CA and SJ sections)

*Leptolepidites* cf. *crassibalteus* Filatoff, 1975 (CA section)

*Leptolepidites proxigranulatus* (Brenner, 1963) Dörhöfer, 1979 (CA section)

*Leptolepidites* spp. (AP, CA and SJ sections)

cf. *Leptolepidites* sp. (SJ section)

*?Lusatisporites dettmannae* (Drugg, 1967) Srivastava, 1972 (AP, CA and SJ sections)

*Microfoveolatosporis pseudoreticulatus* (Hedlund, 1966) Singh, 1983 (CA section)

*Murospora* sp (AP and CA sections)

*Patellasporites tavaredensis* Groot and Groot, 1962 (AP, CA and SJ sections)

*Peromonolites allenensis* Brenner, 1963 (AP, CA and SJ sections)

*Polypodiisporonites cenomanianus* (Singh, 1983) Ravn and Witzke, 1995 (CA section)

cf. *Psilatriletes circumundulatus* Brenner, 1963 (SJ section)

*Reticulosporis gallicus* Deák and Combaz, 1968 (CA section)

*Striatella* cf. *balmei* Filatoff and Price, 1988 (CA section)

*Tigrisporites scurrandus* Norris, 1967 (SJ section)

*Tigrisporites* sp. (CA section)

*Undulatisporites* cf. *pflugii* Pocock, 1970 (CA section)

*Undulatisporites* cf. *rugulatus* Stone, 1973 (AP section)

*Undulatisporites undulapolus* Brenner, 1963 (CA section)

*Undulatisporites* spp. (AP, CA and SJ sections)

*Varirugosisporites tolmanensis* Srivastava, 1972 (CA section)

*Varirugosisporites* sp. (CA section)

*Verrucatosporites* sp. (AP and CA sections)

*Verrucosisporites* cf. *rotundus* Singh, 1964 (AP and CA sections)

*Verrucosisporites* spp. (AP and CA sections)

**Gymnosperm pollen**

Caytoniales

*Vitreisporites pallidus* (Reissinger, 1950) Nilsson, 1958 (AP, CA and SJ sections)

Cycadales/Ginkgoales/Bennettitales

*Cycadopites carpentieri* (Delcourt and Sprumont, 1955) Singh, 1964 (CA section)

*Cycadopites follicularis* Wilson and Webster, 1946 (AP, CA and SJ sections)

*Cycadopites* spp. (AP, CA and SJ sections)

*Monosulcites minimus* Cookson, 1947 ex Couper, 1953 (AP, CA and SJ sections)

*Monosulcites* sp. 1 (AP and SJ sections)

*Monosulcites* spp. (AP, CA and SJ sections)

Coniferales Araucariaceae

*Araucariacites australis* Cookson, 1947 (AP, CA and SJ sections)

*Balmeiopsis limbata* (Balme, 1957) Archangelsky, 1977 (AP, CA and SJ sections)

*Brachyphyllum* cf. *obesum* Heer, 1881 (SJ section)

*Callialasporites dampieri* (Balme, 1957) Dev, 1961 emend. Norris, 1969 (AP, CA and SJ sections)

*Callialasporites minor* Pocock, 1970 (CA section)

*Callialasporites trilobatus* (Balme, 1957) Dev, 1961 (AP and CA section)

*Callialasporites turbatus* (Balme, 1957) Schulz, 1967 (CA section)

*Callialasporites segmentatus* (Balme, 1957) Srivastava, 1963 (AP section)

*Dammarites* cf. *albens* Presl in Sternberg, 1838 (AP section)

*Dammarites* sp. (SJ section)

*Uesuguipollenites callosus* Dino, 1994 (AP, CA and SJ sections)

Coniferales Cheirolepidiaceae

*Classopollis major* Groot and Groot, 1962 (AP, CA and SJ sections)

*Classopollis* cf. *obidosensis* Groot and Groot, 1962 (AP, CA and SJ sections)

*Classopollis* spp. (AP, CA and SJ sections)

*Frenelopsis justae* Barral, Gomez, Daviero-Gomez, Lécuyer,Mendes et Ewin 2019 (SJ section)

*Frenelopsis turolensis* Gomez, 2002 (AP section)

*Frenelopsis* cf. *turolensis* Gomez, 2002 (CA section)

Coniferales Cupressaceae

*Inaperturopollenites dubius* (Potonié and Venitz 1932) Thomson and Pflug 1953 (AP, CA and SJ sections)

*Taxodiaceaepollenites hiatus* (Potonié 1931) Kremp 1949 (AP, CA and SJ sections)

*Perinopollenites halonatus* Paden Phillips and Felix 1971 (AP and CA sections)

*Widdringtonites* sp. (SJ section)

Coniferales Miroviaceae

*Mirovia gothanii* Gomez, 2002 (AP and CA sections)

Coniferales Pinaceae/Podocarpaceae

*Cedripites* cf. *canadensis* Pocock, 1962 (SJ section)

*Cedripites* cf. *cretaceous* Pocock, 1962 (CA section)

*Cedripites* sp. (AP and CA sections)

cf. *Phyllocladidites inchoatus* (auct. non Pierce) Norris, 1967 (SJ section)

*Phyllocladidites* spp. (AP and CA sections)

*Pinuspollenites* spp. (AP, CA and SJ sections)

*Podocarpidites* spp. (AP, CA and SJ sections)

Erdtmanithecales

*Eucommiidites minor* Groot and Penny, 1960 (AP, CA and SJ sections)

*Eucommiidites troedsonii* Erdtman, 1948 (AP, CA and SJ sections)

*Eucommiidites* sp. (CA section)

Ginkoales

*Eretmophyllum* sp. (SJ section)

*Nehvizdya* (=*Eretmophyllum*) *penalveri* Gomez 2000 (AP and CA sections)

Gnetales Ephedraceae

*Distachyapites* sp. (SJ outcrop)

*Equisetosporites barghoornii* Pocock, 1964 (CA section)

*Equisetosporites* *multicostatus* (Brenner, 1963) Norris, 1967 (AP and CA sections)

*Equisetosporites* aff. *rousei* Pocock, 1964 (SJ outcrop)

*Equisetosporites* spp. (AP, CA and SJ sections)

*Gnetaceaepollenites oreadis* Srivastava, 1968 (AP section)

*Steevesipollenites* sp. (AP and CA sections)

Uncertain affinities

*Abietinaepollenites* sp. (SJ section)

*Afropollis jardinus* (Brenner, 1968) Doyle, Jardiné and Doerenkamp, 1982 (AP, CA and SJ sections)

*Alisporites bilateralis* Rouse, 1959 (AP and CA sections)

*Alisporites grandis* (Cookson, 1947) Dettmann, 1963 (AP and CA sections)

*Alisporites* spp. (CA section)

*Cerebropollenites macroverrucosus* (Thiergart, 1949) Schulz 1967 (AP and CA sections)

*Exesipollenites tumulus* Balme, 1957 (AP, CA and SJ sections)

*Exesipollenites* sp. (AP, CA and SJ sections)

*Inaperturopollenites* spp. (AP, CA and SJ sections)

*Parvisaccites amplus* Brenner, 1963 (SJ section)

*Parvisaccites radiatus* Couper, 1958 (AP and CA sections)

*Parvisaccites* spp. (AP and CA sections)

aff. *Parvisaccites* spp. (SJ section)

*Rugubivesiculites* sp. (CA section)

*Sciadopityspollenites?* sp. (AP and SJ sections)

**Angiosperm pollen**

Chloranthaceae

*Asteropollis asteroides* Hedlund and Norris, 1968 (CA section)

*Clavatipollenites hughesii* Couper, 1958 (AP, CA and SJ sections)

*Clavatipollenites tenellis* Paden Phillips and Felix, 1971 (AP, CA and SJ sections)

*Clavatipollenites* cf. *tenellis* Paden Phillips and Felix, 1971 (CA section)

*Clavatipollenites* spp. (AP, CA and SJ sections)

*Hammenia fredericksburgensis* (Hedlund and Norris, 1968) Ward 1986 (AP, CA and SJ sections)

Chloranthaceae-*Ceratophyllum* clade

*Pennipollis escuchensis* Villanueva-Amadoz, Pons, Díez, Ferrer and Sender, 2010 (AP, CA and SJ sections)

*Pennipollis* cf. *peroreticulatus* (Brenner, 1963) Friis, Pedersen and Crane, 2000 (AP, CA and SJ sections)

*Pennipollis reticulatus* (Brenner, 1963) Friis, Pedersen and Crane, 2000 (AP, CA and SJ sections)

*Pennipollis* spp. (AP, CA and SJ sections)

*Transitoripollis anulisulcatus* Góczán and Juhász, 1984 (AP, CA and SJ sections)

*Transitoripollis similis* Góczán and Juhász, 1984 (AP and CA sections)

*Transitoripollis* cf. *similis* Góczán and Juhász, 1984 (SJ section)

*Transitoripollis* sp. (AP, CA and SJ sections)

*Tucanopollis crisopolensis* (Regali, Uesugui and Santos, 1974) Regali, 1989 (CA and SJ sections)

*Tucanopollis* spp. (CA section)

Laurales

“*Liliacidites*” *minutus* (Brenner, 1963) Walker and Walker, 1984 (AP, CA and SJ sections)

Probable monocots

*Liliacidites* cf. *clavatus* Singh, 1971 (AP and CA sections)

*Liliacidites* cf. *kiowaensis* Ward, 1986 (CA section)

*Liliacidites tectatus* Singh, 1983 (AP and CA sections)

*Liliacidites* spp. (AP, CA and SJ sections)

*Monocolpopollenites* sp. (AP, CA and SJ sections)

Eudicots

*Artiopollis praecox* Ward, 1986 (AP and CA sections)

*Cupuliferoidaepollenites parvulus* (Groot and Penny, 1960) Dettmann, 1973 (CA section)

*Cupuliferoidaepollenites* sp. (AP, CA and SJ sections)

*Dryadopollis vestalis* Ward, 1986 (AP and CA sections)

*Foveotricolpites concinnus* Singh, 1971 (CA section)

*Foveotricolpites* sp. (AP-I, AP-II and CA sections)

*Fraxinoipollenites constrictus* (Pierce, 1961) Chlonova, 1976 (AP section)

*Fraxinoipollenites* spp. (AP and CA sections)

*Fraxinoipollenites?* sp. A sensu Barrón et al., 2015 (AP, CA and SJ sections)

*Fraxinoipollenites?* sp. B sensu Barrón et al., 2015 (SJ section)

*Fraxinoipollenites?* sp. (AP section)

cf. *Margocolporites* sp. (CA and SJ sections)

*Phimopollenites* cf. *megistus* Singh, 1983 (SJ section)

*Phimopollenites pseudocheros* Srivastava, 1975 (AP section)

*Phimopollenites pannosus* (Dettmann and Playford, 1968) Dettmann, 1973 (SJ section)

*Retitrescolpites* sp. A sensu Barrón et al., 2015 (AP and SJ sections)

*Retitrescolpites* spp. (CA section)

*Rousea georgensis* (Brenner, 1963) Dettmann, 1973 (AP, CA and SJ sections)

*Rousea* cf. *georgensis* (Brenner, 1963) Dettmann, 1973 (CA section)

*Rousea* cf. *delicata* Ward, 1986 (SJ section)

*Rousea miculipollis* Srivastava, 1975 (AP section)

*Rousea* cf. *prosimilis* (Norris, 1967) Srivastava, 1975 (CA section)

*Rousea* cf. *scitula* Singh, 1983 (CA section)

*Rousea* spp. (AP-II and CA sections)

*Senectotetradites* *grossus* Singh, 1983 (CA section)

*Senectotetradites* sp. (AP and SJ sections)

*Striatopollis* sp. (AP, CA and SJ sections)

*Tetracolpites* sp. (CA section)

*Tricolpites* cf. *amplifissus* (Laing, 1975) Ward, 1986 (AP section)

*Tricolpites* cf. *brnicensis* Palctová, 1971 (AP section)

*Tricolpites* cf. *maximus* (Singh, 1971) Ward, 1986 (AP and CA sections)

*Tricolpites micromunus* (Groot and Penny, 1960) Singh, 1971 (AP, CA and SJ sections)

*Tricolpites minutus* (Brenner, 1963) Dettmann, 1973 (AP and CA sections)

*Tricolpites* cf. *minutus* (Brenner, 1963) Dettmann, 1973 (SJ section)

*Tricolpites nemejci* Pacltová, 1971 (AP, CA and SJ sections)

*Tricolpites* cf. *parvus* Stanley, 1965 (AP, CA and SJ sections)

*Tricolpites sagax* Norris, 1967 (AP and CA sections)

*Tricolpites* cf. *sagax* Norris, 1967 (CA section)

*Tricolpites* *vulgaris* (Pierce, 1961) Srivastava, 1969 (AP section)

*Tricolpites* cf. *vulgaris* (Pierce, 1961) Srivastava, 1969 (AP, CA and SJ sections)

*Tricolpites* sp. cf. *Retitricolpites varireticulatus* Brenner, 1963 (CA and SJ sections)

*Tricolpites* spp. (AP, CA and SJ sections)

*Tricolporoidites* cf. *pacltovae* Ward 1986 (CA section)

*Tricolporoidites* spp. (AP and CA sections)

*Virgo amiantopollis* (Srivastava, 1975) Ward, 1986 (AP and CA sections)

Uncertain affinities

*Crassipollis chaloneri* (Brenner, 1963) Góczán and Juhász, 1985 (AP, CA and SJ sections)

*Crassipollis* sp. (CA section)

*Cretacaeiporites* spp. (AP, CA and SJ sections)

*Dichastopollenites* *dunveganensis* Singh, 1983 (CA and SJ section)

*Dichastopollenites ghazalatensis* Ibrahim, 1996 (CA section)

*Dichastopollenites* cf. *reticulatus* May, 1975 (AP section)

*Dichastopollenites* spp. (AP, CA and SJ sections)

*Doyleipollenites robbinsiae* Ravn and Witzke, 1995 (AP and SJ sections)

*Echinipollis cenomanensis* Pacltová, 1968(CA section)

*Echimonocolpites* sp. (AP section)

*Jusinghipollis* cf. *ticoensis* Llorens and Perez Loinaze, 2016 (AP, CA and SJ sections)

*Montsechia*-type (SJ section)

*Penetetrapites mollis* Hedlund and Norris, 1968 (CA section)

*Retimonocolpites* cf. *crassatus* (Singh, 1971) Singh, 1983 (CA section)

*Retimonocolpites* *dividuus* Pierce, 1961 (AP and CA sections)

*Retimonocolpites* cf. *dividuus* Pierce, 1961 (SJ section)

*Retimonocolpites excelsus* Ward, 1986 (CA section)

*Retimonocolpites* cf. *rotundus* (Kemp, 1968) Juhász and Góczán, 1985 (SJ section)

*Retimonocolpites* cf. *textus* (Norris, 1967) Singh, 1983 (SJ section)

*Retimonocolpites* spp. (AP, CA and SJ sections)

cf. *Serialis* sp. (SJ section)

*Spermatites*-type (AP and CA sections)

*Singhipollis*? *microreticulatus* Juhász and Góczán, 1985 (CA and SJ sections)

*Stellatopollis* cf. *barghoornii* Doyle in Doyle, Van Campo and Lugardon, 1975 (SJ section)

*Stellatopollis* sp. (AP section)

**Arthropoda**

Pseudoscorpiones

Gen. et sp. indet. (San Just amber outcrop)

Acariformes

Family Ametroproctidae:

*Ametroproctus valeriae* Arillo, Subías and Shtanchaeva, 2009 (San Just amber outcrop)

Family Otocepheidae:

*Cretaceobodes martinezae* Arillo, Subías and Shtanchaeva, 2010 (San Just amber outcrop)

Family Scutoverticidae:

*Hypovertex hispanicus* Arillo and Subías, 2016 (San Just amber outcrop)

Family Erythraeidae:

*Leptus* sp. (San Just amber outcrop)

Family Lamellareidae:

*Tenuelamellarea estefaniae* Arillo and Subías, 2016 (San Just amber outcrop)

Family Trhypochthoniidae:

*Trhypochthonius lopezvallei* Arillo, Subías and Shtanchaeva, 2012 (San Just amber outcrop)

Araneae

Family Oonopidae:

*Orchestina* sp. (San Just amber outcrop)

Family Lagonomegopidae:

*Spinomegops aragonensis* Pérez-de la Fuente, Saupe and Selden, 2013 (San Just amber outcrop)

Collembola

Gen. et sp. indet. (San Just amber outcrop)

Archaeaognatha

Gen. et sp. indet. (San Just amber outcrop)

Orthoptera

Gen. et sp. indet. (San Just amber outcrop)

Blattodea

Gen. et sp. indet. (San Just, arroyo de la Pascueta and La Hoya amber outcrops)

Isoptera: Family incertae sedis:

*Aragonitermes teruelensis* Engel and Delclòs, 2010 (San Just amber outcrop)

Mantodea

Family incertae sedis

*Aragonimantis aenigma* Delclòs, Peñalver, Arillo, Engel, Nel, Azar and Ross, 2016 (San Just amber outcrop)

Psocodea

Family Empheriidae:

*Archaeatropos alavensis* Baz and Ortuño, 2000 (San Just and Arroyo de la Pascueta amber outcrops)

*Preempheria antiqua* Baz and Ortuño, 2001 (San Just amber outcrop)

Gen. et sp. indet (San Just and Arroyo de la Pascueta amber outcrops)

Thysanoptera

Family Stenurothripidae:

*Hispanothrips utrillensis* Peñalver and Nel, 2010 (San Just amber outcrop)

Hemiptera

Gen. et sp. indet. (San Just and Arroyo de la pascueta amber outcrops)

Family Perforissidae:

*Iberofoveopsis miguelesi* Peñalver and Szwedo, 2010 (San Just amber outcrop)

Raphidioptera

Gen. et sp. indet. (Arroyo de la Pascueta amber outcrop)

Neuroptera

Family Mantispidae:

*Aragomantispa lacerata* Pérez-de la Fuente and Peñalver, 2019 (San Just amber outcrop)

Coleoptera

Family Ptinidae:

*Actenobius magneoculus* Peris, Philips and Delclòs, 2015 (San Just amber outcrop)

Family Nemonychidae:

*Arra legalovi* Peris, Davis and Delclòs, 2014 (San Just amber outcrop)

Hymenoptera

Gen. et sp. indet. (San Just and Arroyo de la Pascueta amber outcrops)

Family Alavarommatidae:

*Alavaromma orchamum* Ortega-Blanco, Peñalver, Delclòs and Engel, 2011 (San Just amber outcrop)

Family Stigmaphronidae:

*Burmaphron jentilak* Ortega-Blanco, Delclòs and Engel, 2011 (San Just amber outcrop)

Family Gallorommatidae:

*Cretaceomma turolensis* (Ortega-Blanco, Peñalver, Delclòs and Engel, 2011) (San Just amber outcrop)

Family Evaniidae:

*Cretevania alcalai* Peñalver, Ortega-Blanco, Nel and Delclòs, 2010 (San Just amber outcrop)

*Cretevania montoyai* Peñalver, Ortega-Blanco, Nel and Delclòs, 2010 (San Just amber outcrop)

*Cretevania rubusensis* Peñalver, Ortega-Blanco, Nel and Delclòs, 2010 (Arroyo de la Pascueta amber outcrop)

Family Spathiopterygidae:

*Diameneura marveni* Santer and Álvarez-Parra, 2022 (San Just amber outcrop)

*Mymaropsis turolensis* Engel and Ortega-Blanco, 2013 (San Just amber outcrop)

Family Serphitidae:

*Serphites silban* Ortega-Blanco, Delclòs, Peñalver and Engel, 2011 (San Just amber outcrop)

Family Braconidae:

*Utrillabracon electropteron* Álvarez-Parra and Engel, 2022 (San Just amber outcrop)

Diptera

Gen. et sp. indet. (San Just, Arroyo de la Pascueta and La Hoya amber outcrops)

Family Ceratopogonidae:

*Archiculicoides skalskii* (Szadziewski and Arillo, 1998) (San Just amber outcrop)

*Leptoconops zherikhini* Szadziewski and Arillo, 2003 (San Just amber outcrop)

*Protoculicoides hispanicus* Szadziewski and Arillo, 2016 (San Just amber outcrop)

*Protoculicoides sanjusti* Szadziewski and Arillo, 2016 (San Just amber outcrop)

Family Archizelmiridae:

*Burmazelmira grimaldii* Arillo, Blagoderov and Peñalver, 2018 (San Just amber outcrop)

Family Limoniidae:

*Helius turolensis* Kania‑Kłosok, Krzemiński and Arillo, 2021 (San Just amber outcrop)

Family Rhagionidae:

*Litoleptis fossilis* Arillo, Peñalver and García-Gimeno, 2009 (San Just amber outcrop)

Family Dolichopodidae:

*Microphorites utrillensis* Peñalver, 2008 (San Just amber outcrop)

**References of the taxonomic authorities in alphabetical order**

Agasie JM. Late Cretaceous palynomorphs from northeastern Arizona. Micropaleontology. 1969; 15(1): 13–30.

Álvarez-Parra and Engel, 2022 in Álvarez-Parra S, Peñalver E, Delclòs X, Engel MS. A braconid wasp (Hymenoptera, Braconidae) from the Lower Cretaceous amber of San Just, eastern Iberian Peninsula. ZooKeys. 2022; 1103: 65–78.

Archangelsky S. *Balmeiopsis*, nuevo nombre genérico para el palinomorfo *Inaperturopollenites limbatus* Balme. Ameghiniana. 1979; 14: 122–126.

Arillo A, Blagoderov V, Peñalver E. Early Cretaceous parasitism in amber: A new species of Burmazelmira fly (Diptera: Archizelmiridae) parasitized by a Leptus sp. mite (Acari, Erythraeidae). Cretaceous Res. 2018; 86: 24–32.

Arillo A, Peñalver E, García-Gimeno V. First fossil Litoleptis (Diptera: Spaniidae) from the Lower Cretaceous amber of San Just (Teruel Province, Spain). Zootaxa. 2009; 2026: 33–39.

Arillo A, Subías LS, Shtanchaeva U. A new fossil species of oribatid mite, *Ametroproctus valeriae* sp. nov. (Acariformes, Oribatida, Ametroproctidae), from the Lower Cretaceous amber of San Just, Teruel Province, Spain. Cretaceous Res. 2009; 30 (2): 322–324.

Arillo A, Subías LS, Shtanchaeva U. A new genus and species of oribatid mite, *Cretaceobodes martinezae* gen. et sp. nov., from the Lower Cretaceous amber of San Just (Teruel Province, Spain) (Acariformes, Oribatida, Otocepheidae). Paleontol J. 2010; 44 (3): 287–290.

Arillo A, Subías LS, Shtanchaeva U. A new species of fossil oribatid mite (Acariformes, Oribatida, Trhypochthoniidae) from the Lower Cretaceous amber of San Just (Teruel Province, Spain). Syst Appl Acarol. 2012; 17 (1): 106–112.

Arillo and Subías, 2016 in Arillo A, Subías LS, Sánchez-García A. New species of fossil oribatid mites (Acariformes, Oribatida), from the Lower Cretaceous amber of Spain. Cretaceous Res. 2016; 63: 68–76.

Backhouse J. Late Jurassic and Early Cretaceous palynology of the Perth Basin, Western Australia. Bull Geol Surv West Aust. 1988; 135: 1–232.

Balme BE. Plant microfossils from the Lower Triassic of Western Australia. Palaeontology. 1953; 6: 12–40.

Balme BE. Spores and pollen grains from the Mesozoic of Western Australia. CSIRO Coal Res, Tech Commun. 1957; 25: 1–48.

Barral A, Gomez G, Daviero-Gomez V, Lécuyer C, Mendes MM, Ewin TAM. New insights into the morphology and taxonomy of the Cretaceous conifer Frenelopsis based on a new species from the Albian of San Just, Teruel, Spain. Cretaceous Res. 2019; 96: 21–36.

Barrón E, Peyrot D, Rodríguez-López JP, Meléndez N, López del Valle R, Najarro M, Rosales I, Comas-Rengifo MJ. Palynology of Aptian and upper Albian (Lower Cretaceous) amber-bearing outcrops of the southern margin of the Basque-Cantabrian basin (northern Spain). Cretaceous Res. 2015; 52: 292–312.

Baz A, Ortuño VM. Archaeatropidae, a new family of Psocoptera from the Cretaceous amber of Alava, Northern Spain. Ann Entomol Soc Am. 2000; 93 (3): 367–373.

Baz A, Ortuño VM. New genera and species of empheriids (Psocoptera: Empheriidae) from the Cretaceous amber of Alava, northern Spain. Cretaceous Res. 2001; 22 (5): 575–584.

Below WR. Dinoflagellaten-Zysten aus dem oberen Hauterive bis unteren Cenoman Süd-West Marokkos. Palaeontographica Abt B. 1981; 176(1–4): 1–145.

Bint AN. Fossil Ceratiaceae: A restudy and new taxa from the mid-Cretaceous of the Western Interior, U.S.A. Palynology. 1986; 10: 135–180.

Bolkhovitina NA. Spores and pollen characteristic of Cretaceous deposits of central region of USSR. Tr Geol Inst Akad Nauk SSSR, Moscow. 1953; 145(Geol Ser 61): 1–184 (in Russian).

Bolkhovitina NA. Fossil and modern spores of Schizaeaceae. In: Kremp GOW, Spackman W, editors. Catalog of fossil spores and pollen, vol. 25. Pennsylvania: Univ Park; 1961. pp. 1–41.

Bolkhovitina NA. The spores of the fern of the family Gleicheniaceae (taxonomy and distribution). In: Neustardn MI, editor. The importance of palynological analysis for the stratigraphic and paleofloristic investigations. Moscow: Nauka; 1966. pp. 65–75 (in Russian).

Bolkhovitina NA. The spores of the family Gleicheniaceae fers and their importance for the stratigraphy. Tr Geol Inst Akad Nauk SSSR, Moscow. 1968; 186: 1–116 (in Russian).

Brenner GJ. The spores and pollen of the Potomac Group of Maryland. Maryland Dep Geol Mines Water Res Bull. 1963; 27: 1–215.

Brenner GJ. Middle Cretaceous spores and pollen from northeastern Peru. Pollen Spores. 1968; 10: 341–383

Burger D. Palynology of uppermost Jurassic and lowermost Cretaceous strata in the Eastern Netherlands. Leid Geol Meded. 1966; 35: 209–276.

Chlonova AF. Palynological characteristics of Cretaceous deposits at the Kya River (West Siberia). Tr Inst Geol Geofiz Novosibirsk. 1976; 312: 1-102 (In Russian).

Clarke RFA, Verdier J-P. An investigation of microplankton assemblages from the chalk of the isle of Wight, England. Verh Kon Ned Akad Wetenschap, Afdeeling Natuur, Eerste Reeks, 1967; 24(3): 1–96.

Cookson IC. Plant microfossils from the lignites of Kerguelen Archipelago. BANZ Antart Res Exp. 1929–1931, Rep. 1947; A2: 127–142.

Cookson IC. Difference in microspore composition of some samples from a bore at Comaum, South Australia. Aust J Bot. 1953; 1(3): 462–473.

Cookson IC, Dettmann ME. Some trilete spores from the Upper Mesozoic deposits in the Eastern Australian region. Proc R Soc Vic. 1958; 70: 95–138.

Cookson IC, Dettmann ME. On *Schizosporis*, a new form genus from Astralian Cretaceous deposits. Micropaleontology. 1959; 5(2): 213–216.

Cookson IC, Dettmann ME. Reappraisal of the Mesozoic microspore genus *Aequitriradites*. Palaeontology. 1961; 4: 425–427.

Cornet B, Traverse A. Palynological contributions to the chronology and stratigraphy of the Hartford basin in Connecticut and Massachusetts. Geosci man. 1975; 11: 1–33.

Couper RA. Upper Mesozoic and Cainozoic spores and pollen grains from New Zealand. NZ Geol Surv, Paleontol Bull. 1953; 22: 1–65.

Couper RA. British Mesozoic microspores and pollen grains. A Systematic and Stratigraphic Study. Palaeontographica Abt B. 1958; 103(4–6): 75–179.

Davey RJ. Non-calcareous microplankton from the Cenomanian of England, northern France and North America, Part I. Bull Br Mus (Nat Hist) Geol. 1969; 17(3): 103–180.

Davey RJ, Williams GL.. The genus *Hystrichosphaeridium* and its allies. In: Davey RJ, Downie C, Sarjeant WAS, Williams GL, editors. Studies on Mesozoic and Cainozoic dinoflagellate cysts. Bull Br Mus (Nat Hist) Geol Lond; 1966. pp. 53–106.

Davey RJ, Verdier J-P. An investigation of microplankton assemblages from latest Albian (Vraconian) sediments. Rev Esp Micropaleontol. 1973; 5(2): 173–212.

Davies EH. The Anemiacean, Schizaeacean and related spores: an index to genera and species. Can Tech Rep Hydrogr Ocean Sci. 1985; 67: 1–45.

Deák M. Deux nouveaux genres de spore de la série d'argiles et de marnes aptiennes. Földtani Közlöny. 1962; 92(2): 230–235.

Deák M H. Quelques spores striées de l'étage Aptien. Rev Micropaléontol. 1963; 5(4): 251–256.

Deák MH, Combaz A. "Microfossiles organiques" du Wealdien et du Cénomanien dans un sondage de Charente-Maritime. Rev Micropaléontol. 1967; 10(2): 69–96.

Deflandre G. Considérations biologiques sur les microorganismes d’origine planctonique conservés dans les silex de la craie. Bull Biol Fr Belg. 1935; 69: 213–244.

Deflandre G, Courteville H. Note préliminaire sur les microfossiles des silex crétacés du Cambrésis. Bull Soc Fr Microsc. 1939; 8: 95–106.

Delclòs X, Peñalver E, Arillo A, Engel MS, Nel A, Azar D, Ross A. New mantises (Insecta: Mantodea) in Cretaceous ambers from Lebanon, Spain, and Myanmar. Cretaceous Res. 2016; 60: 91–108.

Delcourt AF, Sprumont G. Les spores et grains de pollen du Wealden du Hainaut. Bruxelles: Imprimerie Marcel Hayez; 1955.

Delcourt AF, Dettmann ME, Hughes NF. Revision of some lower cretaceous microspores from Belgium. Palaeontology. 1963; 6(2): 282–292.

Dettmann ME. Upper Mesozoic microfloras from South-Eastern Australia. Proc R Soc Vic. 1963; 77: 1–148.

Dev S. The fossil flora of the Jubalpur. Series 3. Spores and pollen grins. Paleobotanist. 1961; 8(1–2): 43–56.

Dino R. Algumas espécies novas de graos de pólen do Cretáceo Inferior do Nordeste do Brasil. Bol Geociên Petrobrás. 1994; 8(2–4): 257–273.

Döring H. Die sporenpaläontologische Gliederung des Wealden in Westmecklenburg (Strucktur Werle). Geologie. 1965; 47: 1–118.

Döring, Mai, Krutzsch and Schulz, 1963 in Krutzsch W. Atlas der Mittel- und Jungtertiären Dispersen sporen- und pollen- Sowie der Mikroplanktonformen des Nördlichen Mittel-Europas. Lieferung II. Die Sporaen der Anthocerotaceae und der Lycopodiaceae. Abh Zentral Geol Inst. 1963; 2: 1–141.

Dörhöfer G. Distribution and stratigraphic utility of Oxfordian to Valanginian miospores in Europe and North America. Am Assoc Stratigr Palynol Found Contr Ser. 1979; 5b(2): 101–132.

Doyle JA, Jardiné S, Doerenkamp A. *Afropollis*, a new genus of early angiosperm pollen, with notes on the Cretaceous palynostratigraphy and paleoenvironments of northern Gondwana. Bull Centre Rech Explor-Prod Elf Aquitaine. 1982; 6(1): 39–117.

Doyle, 1975 in Doyle JA, Van Campo M, Lugardon B. Observations on exine structure of Eucommiidites and Lower Cretaceous angiosperm pollen. Pollen Spores. 1975; 27(3): 429–486.

Drugg WS. Palynology of the Upper Moreno Formation (Late Cretaceous–Paleocene), Escarpado Canyon, California. Palaeontographica Abt B. 1967; 120: 1–71.

Eisenack A. Mikroplankton aus dem norddeutschen Apt, nebst einigen Bemerkungen über fossile Dinoflagellaten. NJ Geol Paläontol Abh. 1958; 106(3): 383–422.

Engel MS, Delclòs X. Primitive termites in Cretaceous amber from Spain and Canada (Isoptera). J Kansas Entomol Soc. 2010; 83(2): 111–128.

Engel and Ortega-Blanco, 2013 in Engel MS, Ortega-Blanco J, Soriano C, Grimaldi DA, Delclòs X. A new lineage of enigmatic diaprioid wasps in Cretaceous amber (Hymenoptera: Diaprioidea). Am Mus Novit. 2013; 3771: 1–23.

Erdtman G. Did dicotyledonous plants exist in Early Jurassic times? Geol För Stockh Förh. 1948; 70(453): 265–271.

Fauconnier, 2004 in Fauconnier D, Masure E. Les dinoflagellés fossiles. Guide pratique de détermination. Les genres à processus et à archéopyle apical. Orléans: BrGm editions; 2004.

Fensome RA, Williams GL, Barss MS, Freeman JM, Hill JM. Acritarchs and fossil prasinophytes: an index to genera, species and intraspecific taxa. Dallas: Am Assoc Stratigr Palynol; 1990.

Filatoff J. Jurassic Palynology of the Perth Basin, Western Australia. Palaeontographica Abt B. 1975; 154(1–4): 1–133.

Filatoff J, Price PL. A pteridacean spore lineage in the Australian Mesozoic. Mem Assoc Australas Paleontol. 1988; 5: 89–124.

Fradkina AF in Fradkina AF, Kiseleva AV. New palynological data on the precise boundary between the Lower and Upper Cretaceous in central districs of Yaku. Uchen Zap nauchno-issled Inst Geol Arkt (Paleontol Biostratigr). 1964; 4: 65–81 (In Russian).

Friis EM, Pedersen KR, Crane PR. Fossil floral structures of a basal angiosperm with monocolpate, reticulate-columellate pollen from the early Cretaceous of Portugal. Grana. 2000; 39: 226–245.

Góczán F, Juhász M. Monosulcate pollen grains of angiosperms from Hungarian Albian sediments I. Acta Bot Hung. 1984; 30(3–4): 289–319.

Gomez, 2000 in Gomez B, Martín-Closas C, Barale G, Thévenard F. A new species of Nehvizdya (Ginkgoales) from the Lower Cretaceous of the Iberian Ranges (Spain). Rev Palaeobot Palynol. 2000; 111: 49–70.

Gomez B. A new species of Mirovia (Coniferales, Miroviaceae) from the Lower Cretaceous of the Iberian Ranges (Spain). Cretaceous Res. 2002; 23: 761–773.

Gomez, 2002 in Gomez B, Martín-Closas C, Barale G, Solé de Porta N, Thévenard F, Guignard G. Frenelopsis (Coniferales: Cheirolepidiaceae) and related male organ genera from the Lower Cretaceous of Spain. Palaeontology. 2002; 45(5): 997–1036.

Grigorjeva KN. In: Samoilovitch SR, Mtchedlishvili NO, editors. Pollen and spores from Western Siberia (Jurassic–Paleocene). Leningrad: Tr VNIGRI; 1961. pp. 1–77 (in Russian).

Groot JJ, Groot CR. Plant microfossils from Aptian, Albian and Cenomanian deposits of Portugal. Com Serv Geol Portugal. 1962; 46: 133–176.

Groot JJ, Penny JS. Plant microfossils and age of nonmarine Cretaceous sediments of Maryland and Delaware. Micropaleontology. 1960; 6(2): 225–236.

Hedlund RW. Palynology of the Red Branch member of the Woodbine Formation (Cenomanian), Bryan County, Oklahoma. Oklahoma Geol Surv. 1966; 112: 7–69.

Hedlund RW, Norris G. Spores and pollen grains from Frederisksburgian (Albian) strata, Marshall County, Oklahoma. Pollen Spores. 1968; 10: 129–159.

Heer O. Contributions à la flore fossile de Portugal. Lisbonne: Acad Roy Sci; 1881.

Hunt CO. Miospores from the Portland Stone Formation and the lower part of the Purbeck Formation (Upper Jurassic/Lower Cretaceous) from Dorset, England. Pollen Spores. 1985; 27(3–4): 419–451.

Ibrahim MIA. Aptian–Turonian palynology of the Ghazalat-1 Well (GTX-1), Qattara Depression, Egypt. Rev Palaeobot Palynol. 1996; 94: 137–168.

Juhász M. Dispersed Matoniaceae spores from the hungarian lower and middle cretaceous sediments. Acta Biol Szeged. 1979a; 25(1–2): 33–47.

Juhász M. Investigation of some spore genera from the Lower and Middle Cretaceous in Transdanubia. Acta Biol Szeged. 1979b; 25(1–2): 49–64.

Juhász M, Smirnova SB. Gregussisporites orientalis a new spore genus from Albian sediments. Acta Biol Szeged. 1985; 31: 217–219.

Kania-Kłosok I, Krzemiński W, Arillo A. Two new long-rostrum cranefly species from the Cretaceous Iberian amber (Diptera, Limoniidae, *Helius*). Sci Rep. 2021; 11: 12851.

Kedves M. Études palynologiques dans le bassin de Dorog —II— . Pollen Spores. 1961; 3: 101—153.

Kemp EM. Probable angiosperm pollen from the British Barremian to Albian strata. Palaeontology. 1968; 11: 421–434.

Kemp EM. Aptian and Albian miospores from southern England. Palaeontographica Abt B. 1970; 131 (1–4): 73–143.

Kremp G. Pollenanalytische Untersuchung des miozänen Braunkohlenlagers von Konin an der Warthe. Palaeontographica Abt B. 1949; 90(1–3): 53–93.

Krutzsch W. Mikropalàontologische (sporenpalàontoiogische) Untersuchungen in der Braunkohle des Geiseltales. Geol Beih. 1959; 22: 1—425.

Kützing FT. Species algarum. Leipzig: FA Brockhaus; 1849.

Laing JF. Mid-Cretaceous angiosperm pollen from Southern England and Northern France. Palaeontology. 1975; 18(4): 775–808.

Leffingwell HA. 1971. Palynology of the Lance (Late Cretaceous) and Fort Union (Paleocene) formations of the Type Lance, Wyoming. In: Kosanke RM, Cross AT, editors. Symposium on palynology of the Late Cretaceous and the Early Tertiary. Geol Soc Am Spec Pap. 1971; 127: 1–64.

Lentin JK, Williams GL. A monograph of fossil peridinoid dinoflagellate cysts. Bedford Inst Oceanogr Rep Ser. 1976; BI-R-75-16: 1–237.

Llorens M, Pérez Loinaze VS. Late Aptian angiosperm pollen grains from Patagonia: Earliest steps in flowering plant evolution at middle latitudes in southern South America. Cretaceous Res. 2016; 57: 66–78.

Maier D. Planktonuntersuchungen in tertiären und quartären marinen Sedimenten. Ein Beitrag zur Systematik, Stratigraphie und Ökologie der Coccolithophorideen, Dinoflagellaten und Hystrichosphaerideen vom Oligozän bis zum Pleistozän. NJ Geol Paläontol Abh. 1959; 107(3): 278–340.

Markova, 1961 in Ivanova EA, Markova LT. Schizaeaceae. In: Pollen and spores of western Siberia. VNIGRI Plub; 1961. pp. 64–112.

May F. *Dichastopollenites reticulatus*, gen. et sp. nov. – potential Cenomanian guide fossil from southern Utah and northeastern Arizona. J Paleontol. 1975; 49(3): 528–533.

Millioud ME. Dinoflagellates and acritarchs from some western European Lower Cretaceous type localities. In: Brönnimann P, Renz HH, editors. 1st Internatl Conf Planktonic Microfossils. Geneva: EJ Brill; 1969. pp. 420–434.

Newman KR. Upper Cretaceous–Paleocene guide palynomorphs from northwestern Colorado. Univ Colorado Stud. 1965; 2: 1–21.

Nilsson T. Über das Vorkommen eines Mesozoischen Sapropelgesteins in Schonen. Klungl Fysiogr Sallskapets Handl N F. 1958; 69(10): 5–111.

Norris G. Spores and pollen from the Lower Colorado Group (Albian-?Cenomanian) of Central Alberta. Palaeontographica Abt B. 1967; 120(1–4): 72–115.

Norris G. Miospores from the Purbeck Beds and marine Upper Jurassic of Southern England. Palaeontology. 1969; 12(4): 574–620.

Ortega-Blanco J, Delclòs X, Engel MS. Diverse stigmaphronid wasps in Early Cretaceous amber from Spain (Hymenoptera: Ceraphronoidea: Stigmaphronidae). Cretaceous Res. 2011; 32 (6): 762–773.

Ortega-Blanco J, Delclòs X, Peñalver E, Engel MS. Serphitid wasps in early Cretaceous amber from Spain (Hymenoptera: Serphitidae). Cretaceous Res. 2011; 32 (2): 143–154.

Ortega‐Blanco, Peñalver E, Delclòs X, Engel MS. False fairy wasps in early Cretaceous amber from Spain (Hymenoptera: Mymarommatoidea). Palaeontology. 2011; 54 (3): 511–523.

Pacltová B. Some new pollen grains from the Bohemian Cenomanian. Rev Palaeobot Palynol. 1968; 7: 99–106.

Pacltová B. Palynological study of Angiospermae from the Peruc Formation (?Albian-Lower Cenomanian) of Bohemia. Sb Geol Palentol P. 1971; 13: 105–139.

Paden Phillips P, Felix CJ. A study of lower and middle Cretaceous spores and pollen from the southeastern United States. I. Spores. Pollen Spores. 1971; 13(3): 447–473.

Peñalver, 2008 in Arillo A, Peñalver E, Delclòs X. *Microphorites* (Diptera: Dolichopodidae) from the Lower Cretaceous amber of San Just (Spain), and the co-occurrence of two ceratopogonid species in Spanish amber deposits. Zootaxa. 2008; 1920 (1): 29–40.

Peñalver E, Nel P. *Hispanothrips* from Early Cretaceous Spanish amber, a new genus of the resurrected family Stenurothripidae (Insecta: Thysanoptera). Ann Soc Entomol Fr. 2010; 46 (1–2): 138–147.

Peñalver E, Szwedo J Perforissidae (Hemiptera: Fulgoroidea) from the Lower Cretaceous San Just amber (Eastern Spain). Alavesia. 2010; 3: 97–103.

Peñalver E, Ortega-Blanco J, Nel A, Delclòs X. Mesozoic Evaniidae (Insecta: Hymenoptera) in Spanish amber: reanalysis of the phylogeny of the Evanioidea. Acta Geol Sin. 2010; 84 (4): 809–827.

Pérez-de la Fuente R, Peñalver E. A mantidfly in Cretaceous Spanish amber provides insights into the evolution of integumentary specialisations on the raptorial foreleg. Sci Rep. 2019; 9: 13248.

Pérez-de la Fuente R, Saupe EE, Selden PA. New lagonomegopid spiders (Araneae: †Lagonomegopidae) from Early Cretaceous Spanish amber. J Syst Palaeontol. 2013; 11 (5): 531–553.

Peris D, Davis SR, Engel MS, Delclòs X. An evolutionary history embedded in amber: reflection of the Mesozoic shift in weevil-dominated (Coleoptera: Curculionoidea) faunas. Zool J Linn Soc. 2014; 171: 534–553.

Peris D, Philips TK, Delclòs X. Ptinid beetles from the Cretaceous gymnosperm-dominated forests. Cretaceous Res. 2015; 52 (Part B): 440–452.

Pierce RL. Lower Upper Cretaceous plant microfossils from Minnesota. Bull Minnesota Geol Surv. 1961; 42: 1–86.

Playford G. Palynology of Lower Cretaceous (Swan River) strata of Saskatchewan and Manitoba. Palaeontology. 1971; 14(4): 533–565.

Playford G, Dettmann ME. Rhaeto-Liassic plant microfossils from the Leigh Creek Coal Measures, South Australia. Senckenb Lethaea. 1965; 46 (2–3): 127–181.

Pocock SA. Microfloral analysis and age determination of strata at the Jurassic-Cretaceous boundary in the Western Canada Plains. Palaeontographica Abt B. 1962; 111(1–3): 1–95.

Pocock SAJ. Pollen and spores of the Chlamydospermidae and Schizaeaceae form upper Mannville strata of the Saskatoon area. Grana Palynol. 1964; 5(2): 129–209.

Pocock SA. Palynology of the jurassic sediments of Western Canada. Part 1) Terrestrial Species. Palaeontographica Abt B. 1970; 130(1–2): 12–72.

Potonié R. Pollenformen der miozänen Braunkohle. 2. Sitzungsber Ges Naturforch Freund Berlin. 1931; (1–3): 24–27.

Potonié R. Synopsis der Gattungen der Sporae dispersae, I. Teil: Sporites. Beih Geol Jahrb. 1956; 23: 1–103.

Potonié R, Gelletich J. Ueber Pteridophyten-Sporen einer cocänen Braunkohle aus Dorog in Ungarn. Sitzunsber Gess Naturfors Freund Berlin. 1933; 33: 517–528.

Potonié R, Venitz H. Zur Mikrobotanik der miozänen Humodils der Niederrheinischen Bucht. Arb Inst Paläobot Petrogr Brennsteine. 1934; 5: 5–54.

Presl, 1838 in Sternberg KM. Versuch einer geognostich-botanischen Darstellung der Flora der Vorwelt. Vol 2. Leipzig: Deutschen Mus; 1838.

Ravn RL. Miospores from the Muddy Sandstone (upper Albian), Wind River Basin, Wyoming, USA. Paleontographica Abt B. 1995; 234(3–6): 41–91.

Ravn R, Witzke BJ. The palynostratigraphy of the Dakota Formation (?late Albian–Cenomanian) in its type area, Northwestern Iowa and northeastern Nebraska, USA. Palaeontographica Abt B. 1995; 234(3–6): 93–171.

Regali MSP. *Tucanopollis*, um gênero novo das angiospermas primitivas. Bol Geociên Petrobrás. 1989; 3(4): 395–402.

Regali MSP, Uesugui N, Santos AS. Palinologia dos sedimentos meso-cenozóicos do Brasil (II). Bol Tec Petrobrás. 1974; 17(4): 263–301.

Reissinger A. Die "Pollenanalyse" ausgedehnt auf Alle Sedimentgesteine der Geologischen Vergangenheit. Palaeontographica Abt B. 1950; 90: 101–126.

Ross NE. Investigations of the Senonian of the Kristianstad District, S. Sweden. I. On a Cretaceous pollen and spore bearing clay deposit of Scania, a preliminary report. Bull Geol Inst Uppsala. 1949; 34: 25–43.

Rouse GE. Plant microfossils from Kootenay coal-measures strata of British Columbia. Micropaleontology. 1959; 5(3): 303–324.

Santer and Álvarez-Parra, 2022 in Santer M, Álvarez-Parra S, Nel A, Peñalver E, Delclòs X. New insights into the enigmatic Cretaceous family Spathiopterygidae (Hymenoptera: Diaprioidea). Cretaceous Res. 2022; 133: 105128.

Schulz E. Sporenpaläontologische Untersuchungen rëtoliassischer Schichten im Zentralteil des Germanischen Beckens. Paläontol Abh B. 1967; 2(3): 427–633.

Singh C. Microflora of the Lower Cretaceous Mannville group, East-Central Alberta. Res Counc Alberta Bull. 1964; 15: 1–238.

Singh C. Lower Cretaceous microfloras of the Peace River Area, Northwestern Alberta. Res Counc Alberta Bull. 1971; 28 (1): 1–299.

Singh C. Cenomanian microfloras of the Peace River area, northwestern Alberta. Res Counc Alberta Bull. 1983; 44: 1–239.

Skarby A. Revision of *Gleicheniidites senonicus Ross*. Acta Univ Stockholm, Contr Geol. 1964; 11: 59–77.

Srivastava SK. Polospores from Jurassics of Rajasthan, India. Nature, Lond. 1963; 198 (4887).

Srivastava SK. Ephedralean pollen from the Upper Cretaceous edmonton Formation of Alberta, Canada and their paleoecological significance. Can J Earth Sci. 1968; 5: 211–221.

Srivastava SK. Some angiosperm pollen from the Edmonton Formation (Maestrichtian), Alberta, Canada. In: Santapau H, Ghosh AK, Roy SK, Chanda S, Chaudhuri SK, editors. J. Sen Memorial Volume. Calcutta: Bot Soc Bengal; 1969. pp. 47–67.

Srivastava SK. Some spores and pollen from the Paleocene Oak Hill Member of the Naheola Formation, Alabama (U.S.A.). Rev Palaeobot Palynol. 1972; 14: 217–285.

Srivastava SK. Microspores from the Fredericksburg Group (Albian) of the southern United States. Páleobiol Cont. 1975; 6(2): 1–119.

Srivastava, SK.

Stanley EA. Upper Cretaceous and Paleocene plant microfossils and Paleocene dinoflagellates and hystrichosphaerids from northwestern south Dakota. Bull Am Paleontol. 1965; 49(222): 179–347.

Stone JF. Palynology of the Almond Formation (Upper Cretaceous), Rock Springs Uplift, Wyoming. Bull Am Paleontol. 1973; 64(278): 1–135.

Stover LE. *Taurocusporites*, a new trilete spore genus from the Lower Cretaceous of Maryland. Micropaleontology. 1962; 8(1): 55–59.

Stover LE, Evitt WR. Analyses of pre-Pleistocene organic-walled dinoflagellates. Standford: Univ Publ Geol Sci; 1978.

Stover LE, Partridge AD. Tertiary and Late Cretaceous spore and pollen from the Gippsland Basin, Southeastern Australia. Proc R Soc Victoria. 1973; 85: 237–286.

Szadziewski R, Arillo A. Biting midges (Diptera: Ceratopogonidae) from the lower Cretaceous amber from Alava, Spain. Pol Pis Entomol. 1998; 67 (4): 291–298.

Szadziewski R, Arillo A. The oldest fossil record of the extant subgenus *Leptoconops* (Leptoconops) (Diptera: Ceratopogonidae). Acta Zool Cracov. 2003; 46 (suppl.–Fossil Insects): 271–275.

Szadziewski R, Arillo A, Urbanek A, Sontag E. Biting midges of the extinct genus *Protoculicoides* Boesel from Lower Cretaceous amber of San Just, Spain and new synonymy in recently described fossil genera (Diptera: Ceratopogonidae). Cretaceous Res. 2016; 58: 1–9.

Tasch P, McClure K, Oftedahl O. Biostratigraphy and taxonomy of a hystrichosphere - dinoflagellate assemblage from the Cretaceous of Kansas. Micropaleontology. 1964; 10(2): 189–206.

Thiergart F. Der straigraphische Wert mesozoische Pollen und Sporen. Palaeontographica Abt B. 1949; 89: 1–34.

Thomson PW, Pflug HD. Pollen und Sporen des Mitteleuropäischen Tertiärs. Palaeontographica Abt B. 1953; 94: 1–138.

Van Amerom HWJ. Upper-Cretaceous pollen and spores assemblages from the so-called "Wealden" of the province of León (Northern Spain). Pollen Spores. 1965; 7: 93–133.

Vavrdov M. *Trubasporites*, n.fgen., and some other sporomorphs from Carpathian Lower Cretaceous. Casopis Narod Muz Praze. 1964; 133(1): 37–40.

Venkatachala BS, Kar RK, Raza S. Palynology of the Mesozoic sediments of Kutch, W. India - 3. Morphological study and revision of the spore genus Trilobosporites Pant ex Potonié, 1956. The Palaeobotanist. 1968; 17(1–3): 123–126.

Villanueva-Amadoz U, Pons D, Díez JB, Ferrer J, Sender LM. Angiosperm pollen grains of Sant Just site (Escucha Formation) from the Albian of the Iberian Range (north-eastern Spain). Rev Palaeobot Palynol. 2010; 162(3): 362–381.

Walker JW, Walker AG. Ultrastructure of Lower Cretaceous angiosperm pollen and the origin and early evolution of flowering plants. Ann Mo Bot Gard. 1984; 71: 464–521.

Ward JV. Early Cretaceous angiosperm pollen from the Cheyenne and Kiowa Formations (Albian) of Kansas, U.S.A. Palaeontographica Abt B. 1986; 202 (1–6): 1–81.

Weyland H, Greifeld G. Über strukturbietende Blätter unf pflanzliche Mikrofossilien aus den Untersenonen tonen der Gegend von Quedlinburg. Palaeontographica Abt B. 1953; 95 (1–2): 30–52.

Weyland H, Krieger W. Die Sporen und Pollen der Aachener Kreide und ihre bedeutung für die characterisierung des Mittleren Senons. Palaeontographica Abt B. 1953, 95(1–3): 6–29.

Wilson LR, Webster RM. Plant microfossils from a Fort Union coal of Montana. Am J Bot. 1946; 33: 271–278.

White HH. On fossil Xanthidia. Microsc J Lond, 1842; 11: 35–40.
