# Supplementary material for: Biodiversity of ecosystems in an arid setting: The late Albian plant communities and associated biota from eastern Iberia
Source: PLoS One. 2023 Mar 2;18(3):e0282178. doi: 10.1371/journal.pone.0282178 (PMC9980801; doi:10.1371/journal.pone.0282178)
Supplement: Attachment — Submitted filename: Rebuttal_letter.pdf [file pone.0282178.s004.pdf]

To the Academic Editor.

Plos One November 11th, 2022

Dear Dr Enrique Peñalver,

Firstly, we would like to thank you for all the helpful advice and corrections provided with which we fully agree, and we have done our best to meet each and every one of them.

We are also thankful for the constructive comments made by Reviewers 1 and 2 since they have notably improved the quality of our manuscript regarding formal and scientific aspects.

I have listed below the details of how we have tackled and corrected the changes you and both reviewers (1 and 2) kindly suggested according to the different issues you listed in your list of comments. We hope that this new version meets all the requirements you consider necessary for publication.

We look forward to hearing from you and knowing your decision.

Kind regards,

Dr Eduardo Barrón

In the following lines, we have summarised the main changes we have carried out in each section of the manuscript. However, the full record of changes is included in the file titled "Revised_Manuscript_with_track_changes.pdf".

JOURNAL REQUIREMENTS (Editor comments):

1. We have made sure that our manuscript meets PLOS ONE´s style requirements according to the style templates.
2. Regarding the statement about the financial disclosure, we have added the statement you suggested since the funders had no role in the study (see lines 46-48 of the new manuscript).
3. We have added the statement: "The authors have declared that no competing interests exist" as requested (Line 50 of the new manuscript).
4. Regarding the Data availability, we have added the statement: "All relevant data are within the paper and its Supporting Information files" as suggested (see lines 39-40 of the new manuscript).
5. Regarding the issue of figure 1 copyright, we have decided to replace figure 1 (A) with our original figure. This new figure 1(A) has been constructed based on the previous

figure; therefore, we consequently added: “based on data from Aurell et al., 2016” in the figure caption.

6. We have not removed any reference as it was not necessary. However, new references have been added to meet the revisions suggested by Reviewer 2.

Additional Editor Comments:

In figure 17, we try to represent wet interdune environments. Such environments should have been restricted ones showing a very limited lateral extension which is why we represent them as occupying a small portion of the reconstruction carried out in the figure. Conversely, we also represent Araucariaceae-dominated coniferous woodland communities developed closer to the palaeocoast line. These coastal communities are the ones that most likely had formed coastal woodland patches. Finally, we also include the occurrence of xerophytic woodland communities in the reconstruction of Figure 17, which is represented by a few scattered trees.

Besides, we have improved the explanation of the figure (17) caption to be more explanatory than the previous one.

This way we hope that this additional comment has been duly answered.

REVIEWER 1 COMMENTS:

The comments made by Reviewer 1 concerned mostly the abstract and its approach as it seemed to be too specialised for the readership of PLOSONE. Therefore, following the suggestions the reviewer raised, we have changed the abstract to adapt its content to the expertise of a broader readership (see the abstract in the marked-up copy of the manuscript for details). Besides, we have revised the manuscript’s text to avoid very specialised expressions insofar as possible and make our manuscript more suitable for PLOSONE.

The few issues suggested by Reviewer 1 have been most helpful and have contributed to improving the approach of the manuscript.

REVIEWER 2 COMMENTS:

Major questions:

1. Thank you for this suggestion. We believe that Reviewer 2 raised an interesting question. Therefore, we have considered all the references suggested by the reviewer to improve the discussion. In this sense, we have considered the existence of two types of savanna-like communities. One of them mainly consists of ferns of the family Matoniaceae, which was not recorded in the study area of this work as we infer the

occurrence of coniferous woodlands, instead. The difference between both types of fern savannas is indicated in the MS chapter *Environments and vegetation*.

2. Ok, done. We agree with this suggestion. Therefore, we have made a table (Table 1 in the revised MS) as suggested where all the citations of the taxonomic authorities are included. This table combines the information from Appendixes 1 and 2 in an only table that has been included in the main body of the manuscript (instead of being an appendix), as suggested. Besides, all the references of the taxonomic authorities have been added to the S2 Appendix.
3. We could not understand what the reviewer meant in this comment. In the mentioned subchapter we discuss the possibility of pioneer vegetation dominated by ferns, and, according to the reviewer, we dismiss this possibility later in favour of a wet interdune community interpretation. However, we discuss the existence of the fern-dominated pioneer community and the fact that such communities would have developed in the wet interdunes. Therefore, we do not dismiss at all the possibility of a pioneer community dominated by ferns; in fact, it is our interpretation.
4. Ok, thank you for the clarification. It has been corrected in the whole manuscript.
5. Done, thank you. The table (table 3 in the submitted version of the MS) including the arthropod taxa has been moved from "supplementary material" to the main text of the manuscript, which has been named table 2 in the revised version of the MS. This updated table also includes the families of the different arthropod groups identified in amber.

Minor recommendations:

95ff: Ok, thank you. We have rephrased the sentence.

271: Ok, done. As the reviewer suggested, we have changed outcropping to cropping out in the whole text.

613f: We think the discussion of this topic, related to the metabolic evolution of the organisms, is out of the scope of this paper. As the reviewer mentions, it is a very general issue that is not worth discussing in detail. We are simply stating that the identified palaeoflora and palaeofauna assemblages, albeit taphonomically biased in many cases, indicate a high complexity of the ecosystems which shows similarities with their modern counterparts (i.e., Namib and Kalahari Deserts).

629 and elsewhere: Ok, we agree. Following the reviewer´s advice, we have eliminated the colloquial terminology with regard to the families. We changed Cherolepids to Cherolepidiaceae in the whole text.

643, 647: Ok, done. We have removed the “s.l.” (sensu lato) at the end of the name of the families. We have made sure that this has been corrected all over the text.

701: The reviewer is right. We have changed “variegated” to “diversified” as requested.

731ff: Ok, thanks. The sentence has been rephrased as suggested.

884ff: We partly agree with the reviewer in this comment. We have specified in the text the comparison between our assemblages in this work with those from the Basque-Cantabrian Basin. In this sense, we added that both basins´assemblages share about 90 % of the identified families of palaeoflora and a large number of the species. However, we consider that it is out of the scope of this paper to carry out a comparison including percentages of the main genera and families at length.

Regarding the last point, the reviewer mentions: “The Authors observe (if I understand correctly) that the studied palynoflora is similar to those from Gondwana, whereas the megaflora to those from Laurasia. It would be interesting to discuss this observation further”. Our palynoflora is (mostly) from Laurasia; however, we have identified a few palynomorphs with Gondwanan influence since Iberia was in the southern part of Laurasia during the mid-Cretaceous but very close to northern Gondwana. We believe that the reviewer did not understand our idea correctly.

- Table: the mistakes pointed out by the reviewer in Table 1 have been corrected in the new Table 1.
- Figures:
    - Figure 1 has been modified following the suggestions of the Reviewer. (A) represents the Maestrazgo basin and its subdivision in sub-basins as indicated in the figure caption. The yellow colour represents the main depositional areas while the blue areas represent thresholds or areas with low deposition, as the legend of colours of this figure shows. Therefore, this sub-figure is only to locate the sub-figures (B) and (C) that present a detailed geological mapping of the study area as well as to recognise the subdivision of the Maestrazgo Basin into sub-basins. The tectonic structures observed are the modern ones that subdivide the Maestrazgo

Basin; however, these structures coincide with the palaeotectonic guidelines that controlled sedimentation during the Mesozoic as the Iberian Ranges is the result of the tectonic inversion of the Mesozoic intraplate basins of Iberia. Regarding Figures 1 (B) and (C), we have met the reviewer's request and changed the colours of the map to make them more readable. We truly hope that this way this figure is ok.

- Regarding the problem with the pictures of figure 8, 8G presents a darker colour since it probably experienced different diagenesis compared to the rest of the palynomorphs shown in the plate. However, we considered it important to include this palynomorph owing to its biostratigraphic importance.
- Regarding Fig 9AB, this sample is a tetrad; therefore, it has not been possible to focus all the pollen grains it contains simultaneously as they have different depths of field. In the case of the macroflora pictures 11B and 11E, they are dark because the samples are pitch black coloured and it has not been possible to take lighter pictures of these ones.
- Regarding figure 16 the colours that the Reviewer mentions are simply an artistic representation. In Figure 16 there is already a legend of symbols that includes the different palaeoflora groups and families identified which are the relevant information in this figure. Besides, we have included within the figure headings indicating the four environments identified according to what is included in the text.
- Finally, we would like to add that we have uploaded new versions of the following: 1-3, 5-6, 10 since we have made changes in all of them according to the suggestions of the editor and reviewers.
