# Supplementary material for: Biodiversity of ecosystems in an arid setting: The late Albian plant communities and associated biota from eastern Iberia
Source: PLoS One. 2023 Mar 2;18(3):e0282178. doi: 10.1371/journal.pone.0282178 (PMC9980801; doi:10.1371/journal.pone.0282178)
Supplement: Attachment — Submitted filename: Barron.docx [file pone.0282178.s003.docx]

This is a very interesting study, presenting a valuable synthesis (reconstruction of plant palaeocommunities) on the basis of a rich material (mega-, meso-, and microflora as well as insects and sedimentological proxies). Its main value is in dealing with arid ecosystems that are only seldom preserved in the fossil record.

In my opinion the main weakness of the present text is its insufficient taking into account similar studies on other Cretaceous ecosystems. The Authors often refer to Recent plant communities (from New Caledonia, New Zealand, etc.), which is undoubtedly a good thing, but I was astonished not to see references to existing reconstructions of Cretaceous arid ecosystem (see below, 1, for details). The comparisons are sometimes too general to be meaningful (see below, my remarks to lines 884ff); at least the number of common taxa between analysed floras should be given (this is what I meant by “statistic analysis not performed’).

The English language is mostly good, although occasionally there are some mistakes; in one or two cases I was unable to follow. There is also a point related to the rules of nomenclature (below, 4).

My recommendation is that the paper be printed after **moderate revision**. Detailed comments follow, divided into major questions and recommendation to particular points.

**Major questions**

1. The Authors are right in pointing out that arid ecosystems are more rarely preserved than the wet ones. However, the two reff. quoted are quite insufficient, especially in the context of comparisons, insofar as they concern one example from the Jurassic and one from the Tertiary. I recommend in particular the synthetic chapter about the Lower Cretaceous (‘Neocomian’) dune vegetation of the Harz (Mägdefrau, 1968, p. 340–350) with several references. This book contains also a synthetic chapter on the Buntsandstein flora, that might be of some use. A particular type of arid vegetation of the Cretaceous, called a fern savannah, was often quoted (Mohr, 2015; Kvaček, 2017; Halamski *et al.*, 2020), but seldom described in detail. Halamski *et al.* (2020) postulated also the presence of dunes and of a pioneer fern community in the Late Cretaceous (see also below, 3). I would suggest that a more thorough bibliographic research be made and the vegetation described be placed in an over-regional context through a more extensive comparison, especially with other Cretaceous arid ecosystems.
2. I do not agree with systematic palaeontology being relegated to an Appendix. These are basic data and should be treated, if I may say so, with due respect. I propose the Appendix 2 be re-named a Table and inserted into the main text. Moreover, all taxonomic authorities should be quoted in the reference list (see Bénichou L et al., 2022, Joint statement on best practices for the citation of authorities… *Research Ideas and Outcomes* 8:94338).
3. I have a problem with the entire subchapter *Wet interdunes and wetlands* (lines 811–871). The Authors discuss at some length the possibility of a pioneer vegetation dominated by ferns, but finally seem to dismiss this possibility for reasons that are not stated clearly and interpret their vegetation as a kind of climacic wet interdune community. This is not to say that such an interpretation is wrong, but I would like to have a reason given why this interpretation is preferred over the other one. This is also because I have reconstructed (but on the sole basis of microflora) a pioneer fern community (compare Spicer 2003, p. 138) in the Coniacian­–Santonian of Silesia (Halamski *et al.*, 2020), so I’d be simply interested to know whether the Authors think I was wrong or that their community is unrelated to what we have in Silesia (if so, for what reasons). I am not trying to challenge the interpretation but I would just like to see a more detailed argumentation.
4. Please note that according to the ICZN there is a compulsory comma between author and year. For example, not “*Aragomantispa lacerata* Pérez-de la Fuente and Peñalver 2019” but “*Aragomantispa lacerata* Pérez-de la Fuente and Peñalver, 2019”. No such requirement in the ICN.
5. For insects, please provide not only orders, but also families (they are quoted in the text for some taxa and this is an important information).

**Minor recommendations**

95ff: *The Albian vegetation of western Europe has been described in numerous studies describing the micro-, meso- and macroremains [1–7], and highlighting the prevalence of ferns and gymnosperms –mainly conifers (Araucariaceae-Podocarpaceae-Pinaceae- Cupressaceae-Cheirolepidiaceae)–, and the increasing representation of angiosperms.*

Please rephrase. First of all, the beginning of the sentence seems to suggest that the Albian vegetation of Europe is well-known. I do not think this is the case. Secondly, describing… describe. Third, “increasing” – unclear: within the Albian? compared with older floras (if so, which ones)?

271 Please note that *an outcrop* is a name, whereas the verb is *to crop out*. So not “three outcropping successions”, but “three successions cropping out in the studied area” or something like this. Please check in the entire text.

613f: *However, there are no reasons to believe that arid and semi-arid ecosystems from the Cretaceous were less complex than their modern counterparts.*

This is a very far-going affirmation. Personally I am of opinion that there are good reasons to think that Cretaceous ecosystems were somewhat simpler than their modern equivalents, given that the general trend of the biosphere evolution is that of growing complexity (Vermeij, 2019). Perhaps reformulate to avoid discussion on such a general issue, otherwise argument in detail.

629 and elsewhere: What are the ‘cheirolepids’? If you mean the family Cheirolepidaceae, you should say cheirolepidaceans. Similarly, what is ‘taxodioid’ pollen? I propose to use unambiguous Latin names.

643, 647: There seems to be a distinction between *Cupressaceae* and *Cupressaceae* s.l. in the text; this is unclear. Please indicate at the beginning of your text whether you use *Cupressaceae* as including the *Taxodiaceae* or otherwise and then keep consistently to your choice.

701: *Variegated local vegetation*

“Variegated” means having different colours. I do not suppose the Authors wanted to say that. Diversified?

731ff: *The presence of the dolichopodid* Microphorites utrillensis *in amber from the SJ outcrop [135] would support sand-prone coastal habitats for* Araucariacites*-producers as dolichopodids are known to favour this type of habitats.*

“Modern European species of microphorid flies inhabit sandy biotopes.” (Nel et al. 2004, p. 28) So apparently not all dolichopodids are characteristic for sandy habitats, but only a subgroup thereof. Please check and rephrase if needed.

884ff Generally speaking, the palynological comparisons are very imprecise, for example (890ff) “The Maestrazgo palynological assemblages can also be related to the ones described from the Basque-Cantabrian Basin”. I would say everything can be related to everything else, in some way, but what do the Authors want to say precisely?

I would expect that the Authors calculate percentages of common species or genera.

The Authors observe (if I understand correctly) that the studied palynoflora is similar to those from Gondwana, whereas the megaflora to those from Laurasia. It would be interesting to discuss this observation further.

925ff: *Latitudinal effects influenced the composition of the mid-Cretaceous palynological assemblages from Western Europe and the Atlantic Coastal Plain of USA, since they belonged to the Northern Mid-latitude Warm humid belt (NMW belt).*

This is logically flawed. That a flora belongs to a belt is not a datum, but an interpretation. So you cannot say that “the climate influenced the floras, since they belonged…”, but rather “the climate influenced the floras, as evidenced by the fact that floras from similar palaeolatitudes are similar” or something like that.

**Table**

*Foraminisporis assymetricus* SHOULD BE *asymmetricus*

*Camarozonosporites* cf. *hammenii* van Ameron SHOULD BE van Amerom

Why “Monilophyta Anemiaceae” but “Pteridophyta Osmundaceae”? Use either M. or P., but consistently.

**Figures**

Figure 1 should be modified. It is unclear what (A) represents – only Cretaceous strata? Is it a modern tectonic structure or does it correspond (entirely / partly) to a palaeogeographic pattern? This is not explained in the legend. The maps (B) & (C) are illegible because the three variants of the green colour are too similar. Please make them more different from each other.

Palynomorph photographs are mostly of high quality, although 8G is too dark and 9AB slightly out-of-focus. Megaflora photos are less good and 11B & 11E are much too dark (specimens are just black spots).

Fig. 16 is very interesting, but some colours are not explained. In the main figure the topographic surface has six different colours (shades of yellow/beige and green). Moreover, these colours are sometimes different when the same areas are depicted in the main figure and in the enlargements (for example the savannah is dark beige in the marginal zone and light green in the centre on the main figure, but entirely dark beige on the enlargement). Please explain all that.

**Additional references to be taken into account**

Halamski, A.T.,Kvaček, J., Svobodová, M., Durska, E. & Heřmanová, Z. 2020. Late Cretaceous mega-, meso-, and microfloras from Lower Silesia *Acta Palaeontologica Polonica* 65 (4): 811–878.

Kvaček, J 2017. Late Cretaceous Floras in Central Europe and their palaeoenvironment. D.Sc. thesis. DOI: 10.13140/RG.2.2.19586.07361

Mägdefrau K 1968. *Paläobiologie der Pflanzen (vierte Auflage).* Gustav Fischer, Jena.

Mohr B *et al.* 2015. *Ruffordia goeppertii* (Schizaeales, Anemiaceae) A common fern from the Lower Cretaceous Crato Formation of northeast Brazil. *Cretaceous Research*, 54 (2015) 17e26.

Spicer, R.A. 2003. Changing climate and biota. In: P.W. Skelton (ed.), *The Cretaceous World*, 85–162. Cambridge University Press, Cambridge.

Vermeij GJ 2019. Power, competition, and the nature of history. *Paleobiology* 45 (4): 517–530.
